# Optical Genome Mapping for Detection of *BCR::ABL1*—Another Tool in Our Toolbox

**DOI:** 10.3390/genes15111357

**Published:** 2024-10-22

**Authors:** Zhenya Tang, Wei Wang, Gokce A. Toruner, Shimin Hu, Hong Fang, Jie Xu, M. James You, L. Jeffrey Medeiros, Joseph D. Khoury, Guilin Tang

**Affiliations:** 1 Department of Pathology, Microbiology and Immunology, University of Nebraska Medical Center, Omaha, NE 68198, USA; jkhoury@unmc.edu; 2 Department of Hematopathology, The University of Texas MD Anderson Cancer Center, Houston, TX 77030, USA; wwang13@mdanderson.org (W.W.); gatoruner@mdanderson.org (G.A.T.); shu@mdanderson.org (S.H.); hfang@mdanderson.org (H.F.); jxu9@mdanderson.org (J.X.); mjamesyou@mdanderson.org (M.J.Y.); ljmedeiros@mdanderson.org (L.J.M.)

**Keywords:** *BCR::ABL1*, FISH, RT-PCR, optical genome mapping (OGM)

## Abstract

**Background:** *BCR::ABL1* fusion is mostly derived from a reciprocal translocation t(9;22)(q34.1;q11.2) and is rarely caused by insertion. Various methods have been used for the detection of t(9;22)/*BCR::ABL1*, such as G-banded chromosomal analysis, fluorescence in situ hybridization (FISH), quantitative real-time reverse transcription-polymerase chain reaction (RT-PCR) and optical genome mapping (OGM). Understanding the strengths and limitations of each method is essential for the selection of appropriate method(s) of disease diagnosis and/or during the follow-up. **Methods:** We compared the results of OGM, chromosomal analysis, FISH, and/or RT-PCR in 12 cases with *BCR::ABL1*. **Results:** *BCR:ABL1* was detected by FISH and RT-PCR in all 12 cases. One case with ins(22;9)/*BCR::ABL1* was cryptic by chromosomal analysis and nearly missed by OGM. Atypical FISH signal patterns were observed in five cases, suggesting additional chromosomal aberrations involving chromosomes 9 and/or 22. RT-PCR identified the transcript isoforms p210 and p190 in seven and five cases, respectively. Chromosomal analysis revealed additional chromosomal aberrations in seven cases. OGM identified extra cytogenomic abnormalities in 10 cases, including chromoanagenesis and *IKZF1* deletion, which were only detected by OGM. **Conclusions:** FISH offers rapid and definitive detection of *BCR::ABL1* fusion, while OGM provides a comprehensive cytogenomic analysis. In scenarios where OGM is feasible, chromosomal analysis and RT-PCR may not offer additional diagnostic value.

## 1. Introduction

The chimeric *BCR::ABL1* is a diagnostic hallmark of chronic myeloid leukemia (CML), and a recurrent cytogenetic abnormality used for subclassification of B-lymphoblastic leukemia (B-ALL), mixed-phenotype acute leukemia (MPAL), and acute myeloid leukemia (AML) by the 5th edition of the World Health Organization Classification of Hematolymphoid Tumors (WHO-HAEM5) [1,2] and the International Consensus Classification (ICC) guidelines [3,4]. *BCR::ABL1* is mostly derived from a reciprocal t(9;22)(q34.1;q11.2), with the active *BCR::ABL1* located in the derivative chromosome 22, der(22) or the Philadelphia (Ph) chromosome [1,5], whereas the *ABL1::BCR* in the derivative chromosome 9 or der(9) remains inactive. An active *BCR::ABL1* can be formed through the insertion of partial *ABL1* into *BCR* or vice versa [6]. On rare occasions, the *BCR::ABL1* may be located in a chromosome other than der(22) or der(9) [6]. G-banded chromosomal analysis (karyotyping) [7], fluorescence in situ hybridization (FISH) [8], and quantitative real-time PCR [9] are three major methods that have been applied for detecting *BCR::ABL1* fusion in clinical diagnostic laboratories in the past decades.

Karyotyping is usually performed on cultured bone marrow or peripheral blood specimens and detects the *BCR::ABL1* that resulted from t(9;22) by chromosomal morphology [7]. This method also can detect aberrations involving other chromosomes, if any. It usually takes 2–3 days for the cultured specimen to be ready for analysis and 1 to 2 additional days to have a final chromosomal analysis report. The turnaround time (TAT) is usually around 5 workdays or possibly longer. FISH tests often employ fluorescence-labeled DNA probes specifically against *ABL1*, *BCR*, and/or their flanking region(s) [8]. FISH signal pattern(s) will change if an abnormality involving the target(s) (e.g., *ABL1, BCR*) and/or their flanking regions covered by the FISH probes. However, any aberrations beyond the probe-covered regions will not be detected by FISH. A FISH test can be performed on specimens with and/or without cell culture, e.g., aspirate smears, touch prep, and so on. The FISH results can be made ready within 4 h and are definitive for *BCR::ABL1.* A quantitative real-time PCR (or RT-PCR, hereafter) usually applies both *ABL1* and *BCR*-specific primer pairs (even exon-specific for individual isoform type) to amplify *cDNA* synthesized from purified RNA. Quantification is often performed via comparison with an internal control (e.g., *ABL1* level) or external reference material. This method is extremely specific and sensitive and is capable of differentiating the *BCR::ABL1* isoforms [9]. However, it is not capable of detecting aberrations other than a *BCR::ABL1* transcript. Therefore, RT-PCR plays a significant role in monitoring the level of *BCR::ABL1*, especially for a measurable residual disease. Due to the involvement of multiple steps/procedures, such as the extraction of RNA, cDNA synthesis, PCR amplification and detection, and requirement of setting up as many controls as for each step/procedure, a quantitative RT-PCR is usually performed on a batch of specimens that, in return, can impact the TAT of this method.

As a novel powerful technology for detecting both structural variants (SVs) and copy number variants (CNVs) across the whole genome, optical genome mapping (OGM) is becoming popular in clinical diagnostic laboratories. OGM can detect many small but clinically relevant SVs and CNVs beyond the karyotyping detection and, more importantly, provide information on putative fusion genes [10,11,12,13,14,15,16,17,18,19]. In this study, we compare OGM with three other traditional methods (karyotyping, FISH, and RT-PCR) for the detection of *BCR::ABL1* and other cytogenomic alternations in twelve cases with *BCR::ABL1*. We also discuss how these methods should be selected for clinical diagnostic purposes.

## 2. Materials and Methods

### 2.1. Case Selection

This study cohort included 12 cases positive for *BCR::ABL1* that were concurrently evaluated with four methods, karyotyping, FISH, RT-PCR, and OGM, in the Clinical Cytogenetics Laboratory at the University of Texas MD Anderson Cancer Center. The clinicopathologic and laboratory data were collected by electronic medical chart review. This study was approved by the Institutional Review Board and was conducted in compliance with the Declaration of Helsinki.

### 2.2. Chromosomal Analysis

Conventional G-banded chromosomal analysis was routinely performed on unstimulated 24 h and 48 h bone marrow (BM) aspirate and/or peripheral blood (PB) specimens using standard techniques [6,16]. A total of 20 metaphases were analyzed for each case, and the findings were reported according to the International System for Human Cytogenetics Nomenclature (ISCN 2020) [20]. A complex karyotype is defined as ≥3 chromosomal abnormalities with at least one of them being a structural abnormality.

### 2.3. FISH Analysis

FISH analysis was performed primarily on PB or BM smears using the Vysis LSI *BCR/ABL1* ES Dual Color Fusion probe set (referred to as “ES probe”) (Abbott Molecular, Des Plaines, IL, USA). This probe set is designed to distinguish major breakpoints (M-BCR, *p210* isoform) and minor breakpoints (m-BCR, *p190* isoform) when typical signal patterns are observed (2R1G1F for M-BCR and 1R1G2F for m-BCR; R: red, G: green, F: fusion/orange). For cases with atypical signal pattern(s), such as 1R1G1F, which suggested del(9q), a reflexed FISH with Vysis *BCR/ABL1/ASS1* Tri-Color DF FISH probe set (referred to as “tricolor probe”, Abbott Molecular, Des Plaines, IL, USA) was followed [6,16]. Metaphase FISH on G-banded slides was reflexed for cases with complex rearrangements.

### 2.4. Quantitative BCR::ABL1 RT-PCR Assay

A quantitative RT-PCR assay was used routinely to measure *BCR::ABL1* at initial diagnosis and in follow-up studies as described previously [6]. Briefly, 1 µg of total RNA extracted from BM or PB was reversed to cDNA using random primer and superscript II reverse transcriptase (Gibco-BRL) according to the manufacturer’s instructions. The resulting cDNA subsequently underwent amplification with isoform-specific *BCR* and *ABL1* primer sets. The quantitative *BCR::ABL1* mRNA levels are presented as the percent ratio of *BCR::ABL1* to *ABL1* transcript levels. The sensitivity of this assay is between 1 in 10,000 and 1 in 100,000.

### 2.5. Optical Genome Mapping (OGM) Analysis

OGM was performed on fresh PB or BM aspirates using the procedures described previously [16,17,18,19,21,22,23]. Briefly, ultra-high molecular weight (UHMW) genomic DNA (gDNA) was extracted from approximately 1.5 million nucleated cells following the manufacturer’s protocols (Bionano Prep SP-G2 DNA Isolation Kit, Catalog# 90151; Bionano Genomics, San Diego, CA, USA); 750 ng UHMW gDNA was applied for a sequence-specific direct label and stain (Bionano Prep DLS-G2 Labeling Kit, Catalog# 80046). The purified, fluorescence-labeled gDNA molecules were loaded on Saphr G3.3 Chip and then linearized and imaged through massively parallel nanochannel arrays in a Saphr instrument. The Bionano Access software (v1.7) was employed for data analysis, utilizing the Annotated Rare Variant Analysis platform and the Genome Reference Consortium GRCh38/hg38 as the reference genome. The analysis was conducted in two steps, applying two sets of feature files, HemeTargets and hg38-primary_transcripts, along with their corresponding filters. The HemeTargets feature file is custom-designed and encompasses over 500 genes, loci, and fusion genes pertinent to hematologic malignancies. This file was constructed in accordance with guidelines by WHO-HAEM5 [1,2], the International Consensus Classification (ICC) [3,4], National Comprehensive Cancer Network (NCCN) [24,25,26,27], and the National Health Service (NHS) of the UK. Using this file, we screened for critical SVs and CNVs by using the manufacturer (Bionano)-recommended confidence without imposing a minimum size restriction. Concurrently, a 200 Kbp overlap precision was applied to capture gene rearrangements for genes with wide and variable breakpoints. After the initial “hotspot” screening, we shifted to using the hg38-primary_transcripts feature file. For this step, a minimum size of 500 Kbp was set as the filter for both SVs (comprising insertions, deletions, inversions, or duplications) and CNVs.

## 3. Results

### 3.1. Patient Information

This cohort included 12 patients, five men and seven women, aged 36 to 73 years at the time of testing. Seven patients (cases #1–#7) had chronic myeloid leukemia (CML): three at the chronic phase (CP), one at the high-risk chronic phase (CP), and three at the blast phase (BP). Five patients (cases #8–#12) had B-lymphoblastic leukemia (B-ALL) (Table 1). The main-line treatments, patients’ response to treatments (based on NCCN criteria [26,28]), follow-up time, and outcomes are summarized in Table 1. In this cohort, six cases (cases #1, #5, #6, and #8–#10) were initially tested with chromosomal analysis, FISH, and/or RT-PCR as a routine work-up. They were deliberately selected for OGM validation to resent a variety of *BCR::ABL1* positive cases (CML vs. ALL, *p210* vs. *p190 BCR::ABL1* transcripts; cryptic variants, etc.). In the remaining six cases in this cohort, OGM was performed as a clinical diagnostic test per requests by clinicians; after this, a new test was implemented for clinical services.

### 3.2. Chromosomal Analysis Results

**Figure 1:**
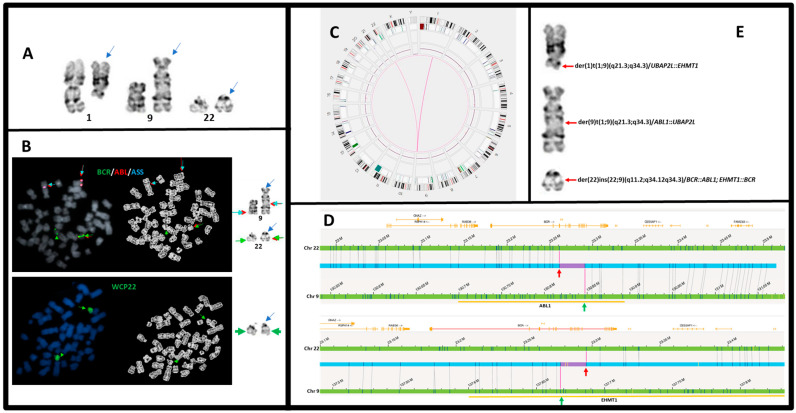
Cytogenetic analyses by three methods in case #6. (**A**) Chromosomal analysis indicated aberrations involving chromosomes 1, 9, and 22 (indicated with blue arrow) and their homologs, respectively. (**B**) FISH analysis using *BCR/ABL1/ASS* tri-color dual-fusion probes indicated neither *BCR* nor *ABL1* signal translocated to chromosome 1 (upper). Whole chromosome painting (wcp) 22 indicated that only the normal and abnormal chromosomes 22 were stained, indicating no chromosome 22 material translocated to chromosomes 1 or 9 (lower). (**C**) Circus plot of OGM showing t(1;9), t(9;22) and del(16q). (**D**) Breakpoints of *BCR* on chromosome 22 (red arrows) and breakpoints of *ABL1* and *EHMT1* on chromosome 9 (green arrows), indicating that the *BCR::ABL1* is derived from an insertion of DNA segment from *3′ABL1* to *5′EHMT1* (about 6.8 Mbp) into *BCR*. (**E**) Details of der(1), der(9), and der(22).

The karyotypic information is summarized in Table 2. Five cases (#1–#5) showed t(9;22) as the sole abnormality, and the other seven cases showed additional abnormalities. Eight patients showed a balanced t(9;22) (#1–#5, #7, #8, and #11), two cases (#10, #12) showed additional Ph chromosome(s), and two cases (#6 and #10) exhibited more complex aberrations involving der(9), der(22), and other chromosomes. One case (#9) had an ins(22;9), which was cryptic for chromosomal analysis. It is worth mentioning that a three-way translocation, t(1;9;22), was reported previously by another institution in case #6 (Figure 1A). However, the der(22) did not resemble a typical Ph chromosome in this case. Instead, our FISH studies on metaphase cells (see below) demonstrated the complexities of chromosomal rearrangements.

### 3.3. FISH Analysis Results

All 12 cases showed high percentages of cells with *BCR::ABL1* (82% to 96.5%) by FISH (Table 2). Cases #2–#3 and #5–#6 showed a signal pattern of 2R1G1F, suggesting a *BCR::ABL1 p210* isoform, while cases #7–#8 and #11 presented a signal pattern of 1R1G2F, indicating a *p190* isoform [6]. The signal patterns in cases #1 and #4 (1R1G1F) and case #9 (2R2G1F) were considered atypical, and they usually indicate additional aberrations involving 9q34 and/or 22q11.2 or a rare mechanism for *BCR::ABL1*, such as insertion (see OGM analysis results below) [6]. The signal patterns in case #10 (2R1G2F and 2R1G3F) and case #12 (1R1G3F and 1R1G2F) were indicative of the presence of two clones with approximately one to two extra copies of the Ph chromosome, which was consistent with chromosomal analysis.

Reflex FISH on previously G-banded metaphases using the *BCR/ABL1/ASS* tri-color probe set on case #6 demonstrated that the *BCR::ABL1* fusion signal was located on the der(22), but there was no FISH signal on chromosome 1 (Figure 1B, upper). Furthermore, whole chromosome painting for chromosome 22 (wcp22) showed that only the normal chromosome 22 and the der(22) were stained (Figure 1B, lower). Therefore, a three-way translocation t(1;9;22) was excluded, and very likely, the *BCR::ABL1* is derived from an insertion of a partial 9q34 containing 3′-*ABL1* in the *BCR* on the der(22) (see OGM analysis below).

### 3.4. Quantitative RT-PCR Analysis Results

Compared with the *ABL1* level in the same specimen, the *BCR::ABL1* fusion expression was high in this cohort; e.g., 31.73% in case #1 and >100% in all other cases (Table 2). The transcript type was the *p210* isoform in seven cases (cases #1–#6, #10) and the *p190* isoform in five other cases. Interestingly, a mixture of *e13a2* and *e14a2* (both encode for *p210* isoform) was detected in three cases (#1–#2, #4).

### 3.5. Optical Genome Mapping (OGM) Analysis Results

**Figure 2:**
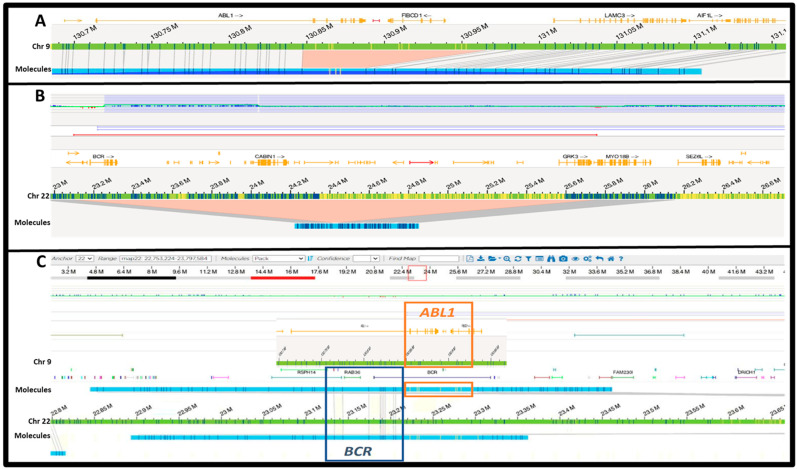
Unusual OGM findings in case #9. (**A**) A deletion involving *3′ABL1* (breakpoint was between exon1 and exon 2) and its flanking region. (**B**) A deletion of about 2.7 Mbp involving *BCR* and its flanking region. However, a gain of 22q11.2 was also observed (the purple bar on the top). (**C**) Manual alignment showing that molecules contained *BCR::ABL1*, derived from an insertion of approximately 100 Kbp fragment containing *3′ABL1* and its flanking region.

A t(9;22)/*BCR::ABL1* rearrangement was called directly by OGM in 11 cases (#1–#8, #10–#12). However, in case #9, OGM did not call a *BCR::ABL1*. Instead, it detected a ~109 Kbp deletion on 9q34 that included partial *ABL1* (breakpoint was between exon 1 and exon 2) and its 3′-flanking region (Figure 2A). Additionally, complex rearrangements including insertion, deletion, and duplication were detected on 22q, which involved *BCR* and its flanking regions (Figure 2B). Manual inspection revealed a fragment exhibiting *BCR::ABL1* recombinant patterns (Figure 2C), suggesting that the *BCR::ABL1* fusion was formed through an ins(22;9) in this case.

In addition to the t(9;22) or ins(22;9) mentioned above, the OGM assay also detected various additional chromosomal abnormalities involving 9q34 and 22q11.2. These included gains of 9q34 and/or 22q11.2 in cases #10 and #12, losses in cases #1 and #4, and complex rearrangements in case #6 (Table 2). As shown in the circos plot, t(1;9) and t(9;22) were detected in case #6 (Figure 1C). Further analysis identified two distinct types of t(9;22): t(9;22)(q34.12;q11.23)/*BCR::ABL1* and t(9;22)(q34.3;q11.23)/*EHMT1::BCR*. The breakpoints at 9q34 involving *ABL1* and *EHMT1*, as indicated by the green arrows (Figure 1D), were spaced approximately 6.8 Mbp apart. When correlated with the FISH results, these data strongly suggest that a DNA fragment of approximately 6.8 Mbp in size, ranging from the 3′*ABL1* to 5′*EHMT1*, was inserted into *BCR* located at 22q11.23. Similarly, the t(1;9) also included two types, t(1;9)/*UBAP2L::EHMT1* and t(1;9)/*ABL1::UBAP2L,* with two breakpoints on 9q34, involving *ABL1* and *EHMT1*. The detailed rearrangements that occurred on der(1), der(9), and der(22) are summarized in Figure 1E.

**Figure 3:**
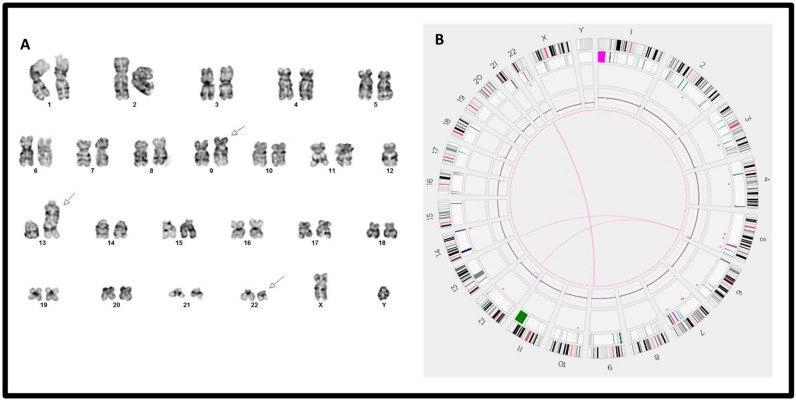
A comparison of chromosomal analysis and OGM assay in case #7. (**A**) Chromosomal analysis indicated a balanced t(9;22)(q34;q11.2) and a psu dic(13;12)(q34;p11.1), as indicated by arrows. (**B**) Circos plot of OGM assay indicated a t(9;22), a t(5;12), and a t(5;13) at the same 5q33.3 band level, an apparent del(12p) and a del(5q) of small size, likely suggesting unbalanced three-way translocation t(5;12;13) in addition to the t(9;22)/*BCR::ABL1* aberration.

**Figure 4:**
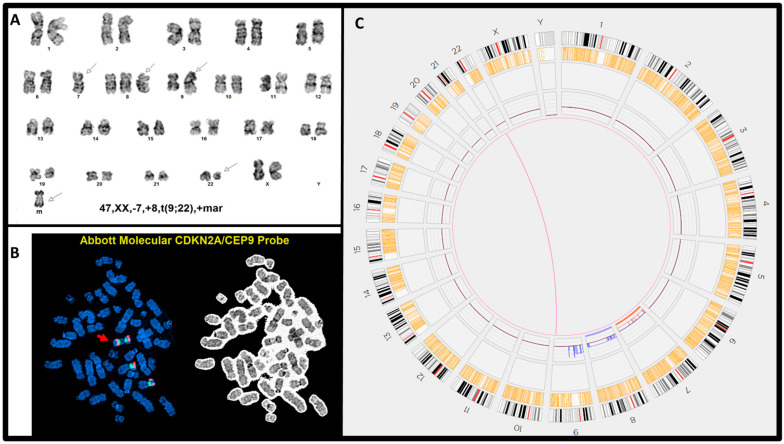
Cytogenetic analyses by three methods in case #8. (**A**) Chromosomal analysis showed a karyotype of 47,XX,-7+8,t(9;22)(q34;q11.2),+mar, indicated by arrows (m: marker chromosome). (**B**) FISH analysis using *CDKN2A/CEP9* probe set showed the marker chromosome containing two copies of *CDKN2A/CEP9* signals (red arrow). (**C**) OGM confirmed all the abnormalities of t(9;22), -7, and +8, as well as four copies of 9p24.3 to q21.11, an idic(9)(q21.11) aberration for the marker chromosome by chromosomal analysis.

The OGM assay also detected multiple chromosomal aberrations beyond the 9q34 and 22q11.2 regions, which were not detected by karyotyping, FISH, or RT-PCR. These included the recurrent abnormalities, del(9p)/*CDKN2A/CDKN2B* loss (cases #10 and #12), del(7p)/*IKZF1* loss (cases #7 and #12), and chromoanagenesis (defined as ≥5 SVs and/or CNVs involving the same chromosome in this cohort) (e.g., cases #9 and #10) (Table 2). Notably, *IKZF1* deletion, a high-risk factor for B-ALL, is often cryptic for chromosomal analysis due to its small size. Multiple non-recurrent SVs and CNVs were detected in single cases (such as in cases #5–#12) (Table 2). For case #7, the chromosomal analysis suggested a karyotype 45,XY,t(9;22)(q34;q11.2),psu dic(13;12)(q34;p11.1)[19]/46,XY[1] (Figure 3A), and OGM detected del(12p), t(5;12), and t(5;13), as well as an interstitial deletion del(5)(q35.1q35.3) (Figure 3B). These findings very likely indicated an unbalanced three-way translocation t(5;12;13), but the alternations in 5q were cryptic for chromosomal analysis. Additionally, the t(5;12)(q33.3;p11.1) resulted in a putative *EBF1::SYT10* gene fusion. For case #8, a karyotype of 47,XX,-7,+8,t(9;22)(q34;q11.2),+mar[20] was detected by chromosomal analysis (Figure 4A). The OGM assay confirmed the presence of t(9;22), -7, and +8, and additionally showed four copies of the 9p24.3 to 9q13 region (Figure 4C), clarifying that the “marker” chromosome was actually idic(9)(q13). This finding was subsequently confirmed by FISH using probes for *CDKN2A* and *CEP9* (Figure 4B).

## 4. Discussion

OGM is a novel technology with a demonstrated power to detect SVs and CNVs throughout the whole genome with high resolution. More and more clinical laboratories are interested in adopting this new technology for patient care. In this study, we performed a head-to-head comparison of OGM versus chromosomal analysis, FISH, and quantitative RT-PCR analyses for the detection of *BCR::ABL1* as well as other cytogenomic aberrations in 12 *BCR::ABL1*+ cases. The latter three methods have been widely applied in clinical laboratory diagnosis for decades. The detection power of each method is listed in Table 3. For detecting *BCR:ABL1*, all four methods can be effective in most cases, although the ins(22;9)/*BCR::ABL1* in case #9 was not detected by karyotyping and OGM at initial analysis due to the small-sized fragment insertion, which is cryptic for chromosomal analysis [6] and complex rearrangements.

**Figure 5:**
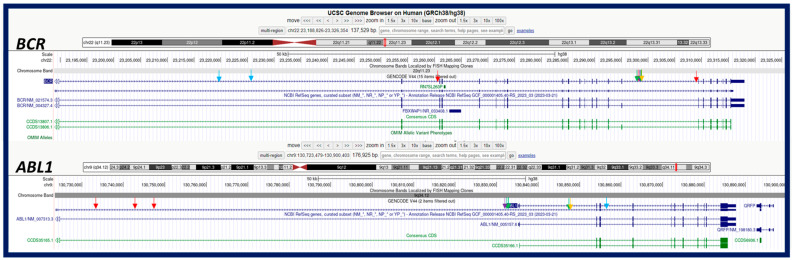
The reported breakpoints of *ABL1* and *BCR* obtained using OGM assay were shown in UCSC Genome Browser in five cases in this cohort (case #1: purple; case #5: green; case #6: orange; case #8: blue; and case #10: red), and their putative isoforms were postulated in each case (Table 3).

The prevalence of *BCR:ABL1* fusion isoforms is different between CML (*p210* predominant) and B-ALL cases (*p190* predominant). Therefore, identification of the isoform type may provide a clue for the differential diagnosis between the CML blast phase and de novo B-ALL at initial diagnosis [1,2,3,4]. It has been reported that a *p190* isoform in CML cases may indicate an inferior response to TKI therapy [29,30,31]. Although both the *e13a2* and *e14a2* subtypes code a *p210* isoform, CML cases with the *e13a2* subtype showed a poorer response to imatinib treatment than that with *e14a2* subtypes [32,33,34]. Therefore, quantitation and determination of the *BCR:ABL1* isoform has become the standard-of-care for all *BCR:ABL1*-positive cases. Among the four methods applied in this cohort, karyotyping was no help in determining the *BCR::ABL1* isoform. In contrast, typical FISH signal patterns may provide clues for *p210* vs. *p190* but cannot distinguish *e13a2* and *e14a2* subtypes of *p210*. As a novel technology, the OGM assay provides both SVs and CNVs with estimated breakpoints at nucleotide (nt) coordinates. The coordinates in six cases in this cohort (Table 3) have been applied to the UCSC GenomeBrowser to map the breakpoints in *BCR* and *ABL1* (Figure 5). The putative *BCR::ABL1* isoforms calculated from these breakpoints involving *BCR* and *ABL1* from OGM data analyses were all *p210*, with other co-existing “unknown” isoforms (e.g., *e1a4* in case #8 and *e3a2* in case #10) (Table 3). Certainly, the putative isoforms from the OGM data were obtained from DNA-level analysis, and they could present differently at the RNA or cDNA levels. In general, putative *BCR::ABL1* isoforms from OGM data should be considered questionable at this stage (Table 3, Figure 5). The RT-PCR using isoform-specific primer pairs is still the gold standard for determining the *BCR::ABL1* isoforms.

Other facts regarding the detection power of each method are also important for an overall evaluation of each case (Table 4). For example, the detection of additional chromosomal aberrations, including additional *BCR::ABL1*, is associated with clonal evolution and the complexity of genome, and thus affects the patient management and outcome [30,31]. The OGM assay has shown the power of this aspect. In addition, the OGM assay detected a few putative novel gene fusions. The rearrangements of *UBAP2L* [35,36] and *EHMT1* [35,36,37] have been reported in liver, lung, breast, ovary, and prostate cancer but not in hematological malignancies, and *ABL1* or *BCR* has not been reported as their partner genes [35,36,37]. Therefore, the function of the novel fusions of *EHMT1::BCR*, *UBAP2L::EHMT1*, and *ABL1::UBAP2L* detected in case #6 remains unknown. Similarly, *EBF1* rearrangement has been reported in B-ALL [38,39,40,41], but was not partnered with *SYT10*, as detected in case #7. The findings of novel fusion genes pave the way for future research into the roles these genetic alterations play in hematological malignancies.

Selecting the appropriate tests from a list of available technologies/platforms currently plays an important role in clinical practice. Several facts need to be considered before making a selection, such as the turnaround time (TAT), sensitivity, and specificity of the method; the purpose of the test (qualitative and/or quantitative determination, further characterization of isoforms, complexity of aberrations, and clonal diversity); and cost-effectiveness (Table 4) [42]. In cases with a high percentage of lymphoblasts, distinguishing the CML blast phase and de novo B-ALL is clinically important but sometimes can be challenging. Screening for Ph+ segmented nuclei on FISH slides can be very helpful for this purpose [2,4,30] and has become a standard procedure in our Clinical Cytogenetics Laboratory.

In summary, we evaluated four testing methodologies, karyotyping, FISH, RT-PCR, and OGM, for their efficacy in detecting *BCR::ABL1* and other cytogenomic aberrations across 12 cases. While OGM effectively identified the *BCR::ABL1* fusion, it had a limitation in potentially missing the fusion if it occurred through the insertion of a very small fragment. Despite this, OGM demonstrated significant advantages by detecting additional aberrations in approximately two-thirds of the cases. These additional findings included novel gene fusions and high-risk cytogenetic aberrations such as chromoanagenesis and *IKZF1* loss, and the latter has a clinical implication in disease progression, prognosis prediction, and modification of therapy regimens in both myeloid and lymphoid neoplasm [43,44,45].

## 5. Conclusions

Our study demonstrated that OGM provides a comprehensive cytogenomic analysis and FISH offers rapid and definitive detection of *BCR::ABL1* fusion. In scenarios where OGM is feasible, chromosomal analysis and RT-PCR may only provide limited diagnostic values.

## Figures and Tables

**Table 1:** The general information of 12 *BCR::ABL1*-positive cases in this study.

Case #	Sex	Age (Year)	Diagnosis	Blast	Treatments	Follow-Up (Month) /Outcome	Outcome
1	M	53	CML-CP	2%	dasatinib, ponatinib, asciminib	6	progression
2	F	36	CML, CP	1%	dasatinib	9	PCyR
3	M	38	CML, CP	2%	ponatinib	12	Died
4	M	66	CML, CP *	15%	ponatinib	4	DMR
5	F	62	CML-BP	90%	imatinib, nilotinib, bosutinib, ponatinib, bosutinib	10	CCyR
6	F	65	CML-BP	93%	dasatinib	10	CCyR
7	M	68	CML-BP	33%	mini-Hyper-CVD, blinatumomab, dasatinib	1	CR
8	F	64	B-ALL, Ph+	90%	blinatumomab, ponatinib.	6	PR
9	F	45	B-ALL, Ph+	85%	blinatumomab, ponatinib.	10	CR
10	M	73	B-ALL, Ph+	95%	blinatumomab, ponatinib.	9	CR
11	F	60	B-ALL, Ph+	67%	mini-Hyper-CVD, blinatumomab, dasatinib	5	CR
12	F	41	B-ALL, Ph+	95%	blinatumomab, ponatinib.	1.5	CR

* High-risk CP. M: male; F: female. CML: chronic myeloid leukemia; CP: chronic phase; BP: blast phase. CCyR: complete cytogenetic response; PCyR: partial cytogenetic response; DMR: deep molecular response. ALL: acute lymphoblastic leukemia. CR: complete remission; PR: partial remission. Ph+: Philadelphia chromosome+.

**Table 2:** A comparison of test results obtained by four different methods in all 12 cases in this study.

Case	Karyotype	FISH Results	RT-PCR (Isoform, Level)	SVs by OGM	CNVs by OGM
1	46,XY,t(9;22)(q34;q11.2)[20]	(ABL1,BCR)x2(ABL1 con BCRx1)[186/200]	e13a2 + e14a2/p210, 31.73%	t(9;22)(q34.12;q11.23)/*BCR::ABL1*	9q34.11q34.12(129120861_130847453)x1
2	46,XX,t(9;22)(q34;q11.2)[20]	(ABL1x3,BCRx2)(ABL1 con BCRx1)[177/200]	e13a2 + e14a2/p210, >100%	t(9;22)(q34.12;q11.23)/*BCR::ABL1*	No
3	46,XY,t(9;22)(q34;q11.2)[20]	(ABL1x3,BCRx2)(ABL1 con BCRx1)[181/200]	e13a2/p210, >100%	t(9;22)(q34.12;q11.23)/*BCR::ABL1*	No
4	46,XY,t(9;22)(q34;q11.2)[20]	(ABL1,BCR)x2(ABL1 con BCRx1)[184/200]	e13a2 + e14a2/p210, >100%	t(9;22)(q34.12;q11.23)/*BCR::ABL1*	9q34.11q34.12((128703248_130135561)x1 22q11.23q12.1(23592450_26715684)x1
5	46,XX,t(9;22)(q34;q11.2)[20]	(ABL1x3,BCRx2)(ABL1 con BCRx1)[182/200]	e13a2/p210, >100%	t(9;22)(q34.12;q11.23)/*BCR::ABL1*	2p12p11.2(80212101_84371148)x1
6	46,XX,der(1)t(1;9)(q21;q34),der(9)t(1;9)(q21;q34),der(22)ins(22;9)(q11.2;q34q34)[20]	(ABL1x3,BCRx2)(ABL1 con BCRx1)[186/200]	e13a2/p210, >100%	t(9;22)(q34.12;q11.23)/*BCR::ABL1* t(1;9)(q21.3;q34.12)/*UBAP2L::ABL1* t(1;9)(q21.3;q34.3)/*UBAP2L::EHMT1*	16q11.1q11.2(38277017_46457433)x1
7	45,XY,t(9;22)(q34;q11.2),psu dic(13;12)(q34;p11.1)[19]/46,XY[1]	(ABL1,BCR)x3(ABL1 con BCRx2)[150/200]	e1a2/p190, >100%	t(9;22)(q34.12;q11.23)/*BCR::ABL1* t(5;12)(q33.3;p11.1)/*EBF1::SYT10* t(5;13)(q33.3;q34)	5q35.1q35.3(172452581_181472714)x1 7p12.2p12.2(50348483_50399656)x1 12p13.2p11.1(11630322_33424504)x1
8	47,XX,-7,+8,t(9;22)(q34;q11.2),+mar[20]	(ABL1,BCR)x3(ABL1 con BCRx2)[189/200] (CDKN2A,CEP9)x4[194/200]	E1a3/p190, >100%	t(9;22)(q34.12;q11.23)/*BCR::ABL1*	(7)x1 (8)x3 9p24.3q13(14566_64960054)x4
9	46,XX,r(7)[19]/46,XX,del(7)(q11.2q32)[1]	(ABL1,BCR)x3(ABL1 con BCRx1)[164/200] (D7Z1x2,D7S522x1)[92/200]	e1a2/p190, >100%	ins(22;9)(q11.23;q34.12q934.12)/*BCR::ABL1* chromoanagenesis (7)	Numerous segmental loss on chr7
10	48,XY,del(6)(q13q23),der(9)del(9)(p13)t(9;22)(q34;q11.2),+21,der(22)t(9;22),+der(22)t(9;22)[9]/48~49,idem,+der(22)t(9;22),+mar[cp9]/46,XY[2]	(ABL1x5,BCRx4)(ABL1 con BCRx3)[118/200]/ (ABL1x4,BCRx3)(ABL1 con BCRx2)[75/200]	e14a2/p210, >100%	t(9;22)(q34.12;q11.23)/*BCR::ABL1* chromoanagenesis (7,8)	6p25.3q14.1(76216_77720354)x3 6q14.1q21(77721612_105275332)x1 8q12.3q24.3(64448847_140421018)x3 9p24.3p21.1(14566_31847914)x1 9p21.1p13.1(31858561_38843343)x0 9q34.12q34.3(130755223_138334464)x4 15q14q15.3(33563820_43499262)x1,(21)x3 22q11.21q11.23(18746350_23133605)x4
11	46,XX,t(9;22)(q34;q11.2)[10]/46,idem,del(20)(q11.2q13.1)[8]/46,idem,t(X;6)(q22;p23)[2]	(ABL1,BCR)x3(ABL1 con BCRx2)[184/200]	e1a2/p190, >100%	t(9;22)(q34.12;q11.23)/*BCR::ABL1* t(X;6)(q22.1;p24.1)/*TRMT2B* t(3;15)(p25.2;q11.2)/*RAF1* fus(6;6)(p24.3;p22.3)	17q22q25(57433782_83246392)x3 20q11.23q13.33(31182877_61861320)x1
12	46,XX,der(8;9)(q10;q10),t(9;22)(q34;q11.2),+der(22)t(9;22)[20]	(ABL1,BCR)x4(ABL1 con BCRx3)[177/200]/ (ABL1,BCR)x3(ABL1 con BCRx2)[16/200]	e1a2/p190, >100%	t(9;22)(q34.12;q11.23)/*BCR::ABL1*	7p12.2p12.2(50273770_50399656)x1 8p23.3p11.2(61805_42571510)x1 9p24.3p12(14566_39591818)x1 9q34.12q34.3(130777258_138334464)x3 22p11.1q12.1(14545087_25515764)x3

SVs: structural variants; CNVs: copy number variants.

**Table 3:** Detailed information of OGM findings regarding *ABL1* and *BCR* or chromosomes 9 and 22 in five cases in this study.

Case #	Aberrations	Chr. Involved *		Breakpoints of #1 Chr.		Breakpoints of #2 Chr.		Orientation	Confidence **	VAF	Putative Gene Fusion	Putative *BCR::ABL1* Isoform	Self Molecule Counts	ISCN
		#1	#2	Breakpoint (bp)	Position in Gene	Breakpoint (bp)	Position in Gene							
1	transl._interchr.	9	22	129,130,483	*PTPA: intron 4*	23,295,730	*BCR1: intron 15*	+/+	**0.87**	0.38	*PTPA::BCR*	*N/A*	84	ogm[GRCh38] t(9;22)(q34.11;q11.23)
	transl._interchr.	9	22	130,832,617	*ABL1: intron 1*	23,295,730	*BCR1: intron 15*	+/+	0.98	0.53	*ABL1::BCR*	*e14a2/p210*	84	ogm[GRCh38] t(9;22)(q34.12;q11.23)
	deletion	9	9	129,120,861	*PTPA: intron 1*	130,847,453	*ABL1: intron 1*	N/A	0.99	0.44	*-*	*N/A*	80	ogm[GRCh38] 9q34.11q34.12(129120861_130847453)x1
5	transl._interchr.	9	22	130,836,231	*ABL1: intron 1*	23,295,730	*BCR1: intron 15*	+/+	1	0.5	*ABL1::BCR*	*e14a2/p210*	83	ogm[GRCh38] t(9;22)(q34.12;q11.23)
	transl._interchr.	9	22	130,847,453	*ABL1: intron 1*	23,295,730	*BCR1: intron 15*	+/+	**0.84**	0.44	*ABL1::BCR*	*e14a2/p210*	77	ogm[GRCh38] t(9;22)(q34.12;q11.23)
6	transl._interchr.	9	1	137,686,151	*EHMT1: intron 1*	154,225,651	*UBAP2L: intron 2*	+/+	0.95	0.28	*UBAP2L::EHMT1*	*N/A*	72	ogm[GRCh38] t(1;9)(q21.3;q34.3)
	transl._interchr.	9	1	137,732,949	*EHMT11: intron 4*	154,225,651	*UBAP2L: intron 2*	+/+	0.97	0.28	*UBAP2L::EHMT1*	*N/A*	95	ogm[GRCh38] t(1;9)(q21.3;q34.3)
	transl._interchr.	9	1	130,836,231	*ABL1: intron 1*	154,231,851	*UBAP2L: intron 4*	+/+	0.98	0.51	*UBAP2L::ABL1*	*N/A*	128	ogm[GRCh38] t(1;9)(q21.3;q34.12)
	transl._interchr.	9	22	130,847,453	*ABL1: intron 1*	23,261,125	*BCR1: intron 3*	+/+	0.98	0.63	*ABL1::BCR*	*E2a2*	110	ogm[GRCh38] t(9;22)(q34.12;q11.23)
	transl._interchr.	9	22	137,667,566	*EHMT1: intron 4*	23,295,730	*BCR1: intron 15*	+/+	**0.89**	0.48	*EHMT1::BCR*	*N/A*	114	ogm[GRCh38] t(9;22)(q34.3;q11.23)
8	transl._interchr.	9	22	130,847,453	*ABL1: intron 1*	23,225,934	*BCR1: intron 1*	+/+	0.99	0.59	*ABL1::BCR*	*e2a2/p210*	122	ogm[GRCh38] t(9;22)(q34.12;q11.23)
	transl._interchr.	9	22	130,855,697	*ABL1: intron 3*	23,219,177	*BCR1: intron 1*	+/+	0.96	0.61	*ABL1::BCR*	*e1a4/?*	88	ogm[GRCh38] t(9;22)(q34.12;q11.23)
10	transl._interchr.	9	22	130,732,573	*ABL1: intron 1*	23,295,730	*BCR1: intron 15*	+/+	**0.93**	0.48	*ABL1::BCR*	*e14a2/p210*	64	ogm[GRCh38] t(9;22)(q34.12;q11.23)
	transl._interchr.	9	22	130,743,975	*ABL1: intron 1*	23,261,125	*BCR1: intron 3*	+/+	0.98	0.34	*ABL1::BCR*	*e3a2/?*	187	ogm[GRCh38] t(9;22)(q34.12;q11.23)
	transl._interchr.	9	22	130,747,294	*ABL1: intron 1*	23,305,888	*BCR1: intron 15*	+/+	**0.6**	0.34	*ABL1::BCR*	*e14a2/p210*	61	ogm[GRCh38] t(9;22)(q34.12;q11.23)

* Chromosomes involved in the aberration; ** the highlighted confidence scores were below our cutoff values as described in the materials and methods; Chr: chromosome or chromosomal; transl._interchr.: translocation_interchromosomal; intrachr_fusion: intrachromosomal fusion; N/A: not applicable.

**Table 4:** A head-to-head comparison of all four methods applied in this study.

	Chr Analysis	FISH	RT-PCR	OGM
**Detection power**				
*BCR::ABL1* fusion	Yes	Yes	Yes	Yes
Isoforms	No	*p210* vs. *p190*	Yes	Questionable
ACAs on der(9)	Yes	Yes	No	Yes
ACAs on der(22)	Yes	Yes	No	Yes
Other ACAs *	Yes	No	No	Yes
Translocation vs. insertion	Likely	Metaphase FISH	No	Likely
Sensitivity	5%	0.5–2%	0.001–0.0001%	10%
Single cell level	Yes	Yes	No	No
**Turn-around time**	3–5 d	4 h	1–7 d	5–7 d
**Cost-effectiveness**	Yes	Yes	Yes	No
**Clinical application**				
Initial diagnosis	Yes	Yes, quick result	Yes, isoform	Yes
Follow-up studies	Yes	Yes	Yes	Not for MRD
Refractory/Relapse	Yes	Yes	Yes	Yes

* chromosome(s) other than der(9) or der(22). Chr: chromosomal; ACAs: additional chromosomal aberration(s); d: day(s). MRD: minimal residual disease.

## Data Availability

The original contributions presented in the study are included in the article, further inquiries can be directed to the corresponding authors.

## References

[1] Khoury J.D., Solary E., Abla O., Akkari Y., Alaggio R., Apperley J.F., Bejar R., Berti E., Busque L., Chan J.K.C. (2022). The 5th edition of the World Health Organization Classification of Haematolymphoid Tumours: Myeloid and Histiocytic/Dendritic Neoplasms. Leukemia.

[2] Alaggio R., Amador C., Anagnostopoulos I., Attygalle A.D., Araujo I.B.O., Berti E., Bhagat G., Borges A.M., Boyer D., Calaminici M. (2022). The 5th edition of the World Health Organization Classification of Haematolymphoid Tumours: Lymphoid Neoplasms. Leukemia.

[3] Arber D.A., Orazi A., Hasserjian R.P., Borowitz M.J., Calvo K.R., Kvasnicka H.M., Wang S.A., Bagg A., Barbui T., Branford S. (2022). International Consensus Classification of Myeloid Neoplasms and Acute Leukemias: Integrating morphologic, clinical, and genomic data. Blood.

[4] Campo E., Jaffe E.S., Cook J.R., Quintanilla-Martinez L., Swerdlow S.H., Anderson K.C., Brousset P., Cerroni L., de Leval L., Dirnhofer S. (2022). The International Consensus Classification of Mature Lymphoid Neoplasms: A report from the Clinical Advisory Committee. Blood.

[5] Arber D.A., Brunning R.D., Le Beau M.M., Falini B., Vardiman J.W., Porwit A., Thiele J., Foucar K., Doehner H., Bloomfield C.D. (2017). Acute Myeloid Leukemia with Recurrent Genetic Abnormalities.

[6] Tang Z., Toruner G.A., Tang G., Cameron Yin C., Wang W., Hu S., Thakral B., Wang S.A., Miranda R.N., Khoury J.D. (2020). Chronic myeloid leukemia with insertion-derived BCR-ABL1 fusion: Redefining complex chromosomal abnormalities by correlation of FISH and karyotype predicts prognosis. Modern Pathol..

[7] Mikhail F.M., Heerema N.A., Rao K.W., Burnside R.D., Cherry A.M., Cooley L.D. (2016). Section E6.1-6.4 of the ACMG technical standards and guidelines: Chromosome studies of neoplastic blood and bone marrow-acquired chromosomal abnormalities. Genet. Med..

[8] Mascarello J.T., Hirsch B., Kearney H.M., Ketterling R.P., Olson S.B., Quigley D.I., Rao K.W., Tepperberg J.H., Tsuchiya K.D., Wiktor A.E. (2011). Section E9 of the American College of Medical Genetics technical standards and guidelines: Fluorescence in situ hybridization. Genet. Med..

[9] Zhen C., Wang Y.L. (2013). Molecular monitoring of chronic myeloid leukemia: International standardization of BCR-ABL1 quantitation. J. Mol. Diagn..

[10] Lestringant V., Duployez N., Penther D., Luquet I., Derrieux C., Lutun A., Preudhomme C., West M., Ouled-Haddou H., Devoldere C. (2021). Optical genome mapping, a promising alternative to gold standard cytogenetic approaches in a series of acute lymphoblastic leukemias. Genes Chromosom. Cancer.

[11] Lühmann J.L., Stelter M., Wolter M., Kater J., Lentes J., Bergmann A.K., Schieck M., Göhring G., Möricke A., Cario G. (2021). The Clinical Utility of Optical Genome Mapping for the Assessment of Genomic Aberrations in Acute Lymphoblastic Leukemia. Cancers.

[12] Neveling K., Mantere T., Vermeulen S., Oorsprong M., van Beek R., Kater-Baats E., Pauper M., van der Zande G., Smeets D., Weghuis D.O. (2021). Next-generation cytogenetics: Comprehensive assessment of 52 hematological malignancy genomes by optical genome mapping. Am. J. Hum. Genet..

[13] Gerding W.M., Tembrink M., Nilius-Eliliwi V., Mika T., Dimopoulos F., Ladigan-Badura S., Eckhardt M., Pohl M., Wünnenberg M., Farshi P. (2022). Optical genome mapping reveals additional prognostic information compared to conventional cytogenetics in AML/MDS patients. Int. J. Cancer.

[14] Rack K., De Bie J., Ameye G., Gielen O., Demeyer S., Cools J., De Keersmaecker K., Vermeesch J.R., Maertens J., Segers H. (2022). Optimizing the diagnostic workflow for acute lymphoblastic leukemia by optical genome mapping. Am. J. Hematol..

[15] Smith A.C., Neveling K., Kanagal-Shamanna R. (2022). Optical genome mapping for structural variation analysis in hematologic malignancies. Am. J. Hematol..

[16] Yang H., Garcia-Manero G., Sasaki K., Montalban-Bravo G., Tang Z., Wei Y., Kadia T., Chien K., Rush D., Nguyen H. (2022). High-resolution structural variant profiling of myelodysplastic syndromes by optical genome mapping uncovers cryptic aberrations of prognostic and therapeutic significance. Leukemia.

[17] Levy B., Baughn L.B., Akkari Y., Chartrand S., LaBarge B., Claxton D., Lennon P.A., Cujar C., Kolhe R., Kroeger K. (2023). Optical genome mapping in acute myeloid leukemia: A multicenter evaluation. Blood Adv..

[18] Valkama A., Vorimo S., Kumpula T.A., Räsänen H., Savolainen E.R., Pylkäs K., Mantere T. (2023). Optical Genome Mapping as an Alternative to FISH-Based Cytogenetic Assessment in Chronic Lymphocytic Leukemia. Cancers.

[19] Vieler L.M., Nilius-Eliliwi V., Schroers R., Vangala D.B., Nguyen H.P., Gerding W.M. (2023). Optical Genome Mapping Reveals and Characterizes Recurrent Aberrations and New Fusion Genes in Adult ALL. Genes.

[20] McGowan-Jordan J., Hastings R.J., Moore S. (2020). ISCN (2020): An International System for Human Cytogenetic Nomenclature.

[21] Brandes D., Yasin L., Nebral K., Ebler J., Schinnerl D., Picard D., Bergmann A.K., Alam J., Köhrer S., Haas O.A. (2023). Optical Genome Mapping Identifies Novel Recurrent Structural Alterations in Childhood ETV6::RUNX1+ and High Hyperdiploid Acute Lymphoblastic Leukemia. Hemasphere.

[22] Soler G., Ouedraogo Z.G., Goumy C., Lebecque B., Aspas Requena G., Ravinet A., Kanold J., Véronèse L., Tchirkov A. (2023). Optical Genome Mapping in Routine Cytogenetic Diagnosis of Acute Leukemia. Cancers.

[23] Wagener R., Brandes D., Jung M., Huetzen M.A., Bergmann A.K., Panier S., Picard D., Fischer U., Jachimowicz R.D., Borkhardt A. (2023). Optical genome mapping identifies structural variants in potentially new cancer predisposition candidate genes in pediatric cancer patients. Int. J. Cancer.

[24] Brown P.A., Shah B., Advani A., Aoun P., Boyer M.W., Burke P.W., DeAngelo D.J., Dinner S., Fathi A.T., Gauthier J. (2021). Acute Lymphoblastic Leukemia, Version 2.2021, NCCN Clinical Practice Guidelines in Oncology. J. Natl. Compr. Cancer Netw. JNCCN.

[25] Wierda W.G., Brown J., Abramson J.S., Awan F., Bilgrami S.F., Bociek G., Brander D., Chanan-Khan A.A., Coutre S.E., Davis R.S. (2022). NCCN Guidelines^®^ Insights: Chronic Lymphocytic Leukemia/Small Lymphocytic Lymphoma, Version 3.2022. J. Natl. Compr. Cancer Netw. JNCCN.

[26] Narlı Özdemir Z., Kılıçaslan N.A., Yılmaz M., Eşkazan A.E. (2023). Guidelines for the treatment of chronic myeloid leukemia from the NCCN and ELN: Differences and similarities. Int. J. Hematol..

[27] Pollyea D.A., Altman J.K., Assi R., Bixby D., Fathi A.T., Foran J.M., Gojo I., Hall A.C., Jonas B.A., Kishtagari A. (2023). Acute Myeloid Leukemia, Version 3.2023, NCCN Clinical Practice Guidelines in Oncology. J. Natl. Compr. Cancer Netw. JNCCN.

[28] Deininger M.W., Shah N.P., Altman J.K., Berman E., Bhatia R., Bhatnagar B., DeAngelo D.J., Gotlib J., Hobbs G., Maness L. (2020). Chronic Myeloid Leukemia, Version 2.2021, NCCN Clinical Practice Guidelines in Oncology. J. Natl. Compr. Cancer Netw. JNCCN.

[29] Verma D., Kantarjian H.M., Jones D., Luthra R., Borthakur G., Verstovsek S., Rios M.B., Cortes J. (2009). Chronic myeloid leukemia (CML) with P190 BCR-ABL: Analysis of characteristics, outcomes, and prognostic significance. Blood.

[30] Chen Z., Hu S., Wang S.A., Konopleva M., Tang Z., Xu J., Li S., Toruner G., Thakral B., Medeiros L.J. (2020). Chronic myeloid leukemia presenting in lymphoblastic crisis, a differential diagnosis with Philadelphia-positive B-lymphoblastic leukemia. Leuk. Lymphoma.

[31] Abdelmagid M.G., Litzow M.R., McCullough K.B., Gangat N., Pardanani A., Murthy H.S., Foran J.M., Ketterling R.P., Viswanatha D., Begna K.H. (2022). Chronic phase CML with sole P190 (e1a2) BCR::ABL1: Long-term outcome among ten consecutive cases. Blood Cancer J..

[32] Mulas O., Caocci G., Annunziata M., Martino B., Luciano L., Castagnetti F., Pregno P., Galimberti S., Albano F., Orlandi E.M. (2020). Favorable outcome of chronic myeloid leukemia co-expressing e13a2 and e14a2 transcripts, treated with nilotinib. Hematol. Oncol..

[33] Marcé S., Xicoy B., García O., Cabezón M., Estrada N., Vélez P., Boqué C., Sagüés M., Angona A., Teruel-Montoya R. (2021). Impact of BCR-ABL1 Transcript Type on Response, Treatment-Free Remission Rate and Survival in Chronic Myeloid Leukemia Patients Treated with Imatinib. J. Clin. Med..

[34] Salmon M., White H.E., Zizkova H., Gottschalk A., Motlova E., Cerveira N., Colomer D., Coriu D., Franke G.N., Gottardi E. (2022). Impact of BCR::ABL1 transcript type on RT-qPCR amplification performance and molecular response to therapy. Leukemia.

[35] Gao Q., Liang W.W., Foltz S.M., Mutharasu G., Jayasinghe R.G., Cao S., Liao W.W., Reynolds S.M., Wyczalkowski M.A., Yao L. (2018). Driver Fusions and Their Implications in the Development and Treatment of Human Cancers. Cell Rep..

[36] Yoshihara K., Wang Q., Torres-Garcia W., Zheng S., Vegesna R., Kim H., Verhaak R.G. (2015). The landscape and therapeutic relevance of cancer-associated transcript fusions. Oncogene.

[37] Hu X., Wang Q., Tang M., Barthel F., Amin S., Yoshihara K., Lang F.M., Martinez-Ledesma E., Lee S.H., Zheng S. (2018). TumorFusions: An integrative resource for cancer-associated transcript fusions. Nucleic Acids Res..

[38] Roberts K.G., Morin R.D., Zhang J., Hirst M., Zhao Y., Su X., Chen S.C., Payne-Turner D., Churchman M.L., Harvey R.C. (2012). Genetic alterations activating kinase and cytokine receptor signaling in high-risk acute lymphoblastic leukemia. Cancer Cell.

[39] Roberts K.G., Li Y., Payne-Turner D., Harvey R.C., Yang Y.L., Pei D., McCastlain K., Ding L., Lu C., Song G. (2014). Targetable kinase-activating lesions in Ph-like acute lymphoblastic leukemia. N. Engl. J. Med..

[40] Gu Z., Churchman M., Roberts K., Li Y., Liu Y., Harvey R.C., McCastlain K., Reshmi S.C., Payne-Turner D., Iacobucci I. (2016). Genomic analyses identify recurrent MEF2D fusions in acute lymphoblastic leukaemia. Nat. Commun..

[41] Fazio G., Bresolin S., Silvestri D., Quadri M., Saitta C., Vendramini E., Buldini B., Palmi C., Bardini M., Grioni A. (2022). PAX5 fusion genes are frequent in poor risk childhood acute lymphoblastic leukaemia and can be targeted with BIBF1120. EBioMedicine.

[42] Akkari Y.M.N., Baughn L.B., Dubuc A.M., Smith A.C., Mallo M., Dal Cin P., Diez Campelo M., Gallego M.S., Granada Font I., Haase D.T. (2022). Guiding the global evolution of cytogenetic testing for hematologic malignancies. Blood.

[43] Eckardt J.N., Stasik S., Röllig C., Petzold A., Sauer T., Scholl S., Hochhaus A., Crysandt M., Brümmendorf T.H., Naumann R. (2023). Mutated IKZF1 is an independent marker of adverse risk in acute myeloid leukemia. Leukemia.

[44] Paolino J., Tsai H.K., Harris M.H., Pikman Y. (2024). IKZF1 Alterations and Therapeutic Targeting in B-Cell Acute Lymphoblastic Leukemia. Biomedicines.

[45] Pieters R., de Groot-Kruseman H., Fiocco M., Verwer F., Van Overveld M., Sonneveld E., van der Velden V., Beverloo H.B., Bierings M., Dors N. (2023). Improved Outcome for ALL by Prolonging Therapy for IKZF1 Deletion and Decreasing Therapy for Other Risk Groups. J. Clin. Oncol..

